# Hemoglobin-mediated lipid oxidation of herring filleting co-products during ensilaging and its inhibition by pre-incubation in antioxidant solutions

**DOI:** 10.1038/s41598-021-98997-4

**Published:** 2021-09-30

**Authors:** Mursalin Sajib, Haizhou Wu, Rikard Fristedt, Ingrid Undeland

**Affiliations:** Food and Nutrition Science, Department of Biology and Biological Engineering, Chalmers University of Technology, 41296 Gothenburg, Sweden

**Keywords:** Biochemistry, Biotechnology

## Abstract

The aims of this study were to investigate the role of hemoglobin (Hb) in lipid oxidation development during ensilaging of herring filleting co-products, and, to inhibit this reaction by pre-incubating the co-products in water or physiological salt, with/without different antioxidants. Results showed that both peroxide value (PV) and 2-thiobarbituric acid reactive substances (TBARS) gradually increased during 7 days of ensilaging at 22 °C in absence of antioxidants. The increase in TBARS was proportional to the Hb levels present, while PV was less affected. A Hb-fortified Tris-buffer model system adjusted to pH 3.50 confirmed that Hb changed immediately from its native oxyHb to the metHb state, which facilitated heme group release and thus probably explains the increased PV and TBARS during ensilaging. Pre-incubating the co-products for 30 s in a solution containing 0.5% rosemary extract was the most promising strategy to inhibit lipid oxidation both in the co-products during pre-processing storage and during the actual ensilaging. The solution could be re-used up to ten times without losing its activity, illustrating that this methodology can be a scalable and cost-effective strategy to extend the oxidative stability of herring co-products allowing for further value adding e.g., into a high-quality silage.

## Introduction

Industrial seafood processing generates roughly 50% (w/w) co-products^1^, which traditionally are used mainly for mink feed or fish meal production. The mink industry has however drastically declined in the past years, and fish meal production is an energy-intensive process requiring large processing facilities and investments. Besides, the co-products contain significant amounts of protein and long chained n-3 polyunsaturated fatty acids (LC n-3 PUFAs) which should be handled as gently as possible to maintain them in an intact and digestible form^2^. Ensilaging—preservation under acidic conditions—is one of the “green” process options which can be used to valorize fish co-products into feed and/or potential food applications^3^. The principle of ensilaging is quite straightforward; minced fish co-products are mixed with organic acids to lower the pH below 4.0 to preserve the co-products against microbial growth and at the same time induce endogenous protease-mediated autolysis, resulting in a product known as silage^4^. The quality of silage depends largely on the quality of the co-product raw material; thus, the latter should be optimized to produce a high-quality “silage 2.0” suitable for both feed and food applications.

Filleting co-products from pelagic fish like herring (*Clupea harengus*) are cheap and good candidates for ensilaging. However, this raw material is highly susceptible to lipid oxidation (i.e. rancidity) owing to its PUFA content and the abundance of blood-derived hemoglobin (Hb)^5^; the latter has been identified as the major lipid pro-oxidant in fish^6^. Hb is a tetramer consisting of two α chains and two β chains, where each chain contains one porphyrin (heme) moiety having an iron atom in the center of the porphyrin ring^7^. There are several mechanisms by which Hb can catalyze lipid oxidation, e.g. Hb autooxidation, where the reduced iron atom (Fe^2+^) of each heme moiety is oxidized to the met state (Fe^3+^) forming methemoglobin (metHb), which weakens the porphyrin-globin linkage resulting in hemin release. The latter decomposes preformed lipid hydroperoxides into free radicals and thus promotes lipid oxidation^7^. Further, the pro-oxidative activity of Hb, assessed in a flounder Hb model system, has been reported to be pH-dependent^8^; that is, a reduction in pH lowers Hb’s affinity for oxygen, via both the Bohr and Root effects, causing Hb deoxygenation which ultimately results in Hb autooxidation and hemin release^7,9^. For example, in a washed cod mince model system containing pollock, mackerel, menhaden, and flounder Hb, an accelerated lipid oxidation was noticed at pH 6.0, compared to pH 7.2, which correlated to higher formation of deoxyHb and metHb^10^. Thus, we hypothesize that the acidic pH used during ensilaging could be a critical factor behind the documented development of lipid oxidation when using Hb-rich fish raw material^11^. However, to date, no studies have reported the role of Hb in lipid oxidation development during ensilaging of Hb-rich fish co-products.

To minimize Hb-mediated lipid oxidation during processing of fish, several strategies have been reported to date, e.g. use of phytic acid to precipitate Hb during pH-shift processing of cod muscle^12^, increasing the pH of fish muscle minces to avoid Hb-deoxygenation and met-Hb formation^10^, and washing of fish mince such as in surimi production^13^. However, these reported strategies are not compatible with e.g. large-scale ensilaging of fish filleting co-products, calling for further developments. Recently we reported incubation or dipping of herring co-products in 0.9% NaCl or water, with and without antioxidants, as ways to minimize Hb-mediated lipid oxidation during subsequent ice storage of the co-products at pH 6.5–7.5^5^. However, the effectiveness of such to minimize lipid oxidation during subsequent ensilaging of herring co-products at pH ~ 3.50 has never been reported, but we hypothesize that the reduction in Hb-levels and introduction of antioxidants would substantially elevate the silage quality.

The aims of this study were to investigate, (i) the role of Hb in lipid oxidation development during ensilaging of herring filleting co-products, and, (ii) the effect of subjecting the co-products to pre-incubation in 0.9% NaCl with and without added antioxidants on lipid oxidation development during subsequent silage production. In addition, the effect of incubation time, solution to co-products ratio, and reuse of the incubation solution for several incubation treatments were investigated to make it a scalable and easily applicable technology.

## Material and methods

### Materials

Herring filleting co-products were obtained from Scandic Pelagic Ellös AB (Ellös, Sweden). Filleting co-products from the same batch were used for different treatments within the same trial. To compensate for the potential influence of batch-to-batch variations on the results from the different sub-trials, non-incubated or water/saline-incubated controls from each raw material batch were included in each sub-trial, so that a direct comparison could be made between treated/non-treated samples, yielding information on the relative difference caused by incubation per se, or by the included antioxidants. Batch 1 co-products were from herring filleted on March 27th 2018, batch 2 from herring filleted on September 4th 2018, batch 3 from herring filleted on October 21st 2019, and batch 4 from herring filleted on October 29th 2019. The filleting co-products—consisting of heads, frames, tails, skins, belly flaps, blood, guts and other intestinal organs—were collected immediately after filleting, packed in a plastic bag, covered with ice, transported to the lab within 2–6 h under cold storage (5 °C), and then subjected to different treatments as described in Table 1. Thereafter the co-products were minced using a meat grinder (la Minerva, Italy) with a 4.5 mm hole plate and ensilaged as described later.

**Table 1:** Treatment solutions and treatment conditions used in different incubation trials.

Trials	Treatment solutions (in w/v units)	Treatment conditions	Co-products batch
1	Tap water, 0.9% NaCl, and 3% NaCl	Incubated for 30 s or 2 h in treatment solutions (5:1 solution to co-products ratio)	Batch 1
2	Tap water, 0.9% NaCl, 5% MANC in tap water, 0.2% isoascorbic acid with 0.044% EDTA in 0.9% NaCl, and 0.2% isoascorbic acid with 0.044% EDTA in tap water	Incubated for 20 min in treatment solutions (5:1 solution to co-products ratio)	Batch 2
3	Tap water, 2% MANC in tap water, 2% isoascorbic acid in tap water	Incubated for 20 min in treatment solutions (5:1 solution to co-products ratio)	Batch 3
4	0.5% rosemary extract in 0.9% NaCl, 2% isoascorbic acid in 0.9% NaCl	Incubated for 30 s in treatment solutions (2:1 and 3:1 solution to co-products ratio)	Batch 4

MANC: Duralox MANC-213 (TPC: 69.63 ± 2.29 mg gallic acid eq/g; carnosic acid: 1.55 ± 0.06 mg/g; Kalsec, Kalamazoo, Mich., UK); *EDTA* ethylenediaminetetraacetic acid; rosemary extract (TPC: 102.53 ± 0.04 mg gallic acid eq/g; carnosic acid: 57.84 ± 1.54 mg/g; Senyuan Bencao Natural Products Co., Ltd., Yuzhou, Henan, China).

### Determination total Hb in herring co-products

Total Hb content in herring filleting co-products (batch 2) was measured according to Hornsey^14^ with slight modifications as described by Harrysson, et al.^15^. Briefly, 18 ml freshly made acidic acetone solution was added to 4 g of minced herring co-products to a final concentration of 80% acetone, 2% HCl, and 18% water. The sample was then incubated at 8 °C for 60 min in darkness, followed by centrifugation at 5000 × *g* for 10 min. The absorbance of the supernatant was then measured at 640 nm, and Hb concentration was calculated using Bovine Hb as the standard (Sigma-Aldrich, USA).

### Ensilaging with added trout hemolysate

To confirm the role of Hb in lipid oxidation development during ensilaging of herring filleting co-products, trout (*Oncorhynchus mykiss)* hemolysate, prepared according to Fyhn, et al.^16^, was added to minced herring co-products (batch 2) at two different levels—double and triple the amount of their respective controls—with the hypothesis that the lipid oxidation level would be proportionally elevated^6^. The Hb concentration in trout hemolysate, measured according to Benesch, et al.^17^, was 336.08 µmole/L; and, the distribution among different forms was: 98.80% oxy, 0.60% deoxy, and 0.60% met. The final Hb concentrations when duplicating and triplicating the Hb-levels were 114.46 and 147.17 µmole Hb/kg silage, respectively. Two controls were prepared by adding the same volumes of Tris buffer (0.1 M, pH 8.0) as the added volumes of trout hemolysate in the samples, resulting in the final Hb concentrations of 57.23 and 49.05 µmole Hb/kg silage, respectively. The co-products, with or without added hemolysate, were then ensilaged as described later. The extent of lipid oxidation was evaluated by oxidation markers peroxide value (PV) and 2-thiobarbituric acid reactive substances (TBARS).

### Ensilaging of minced herring filleting co-products

Ensilaging was performed by adding 2.5% (v/w) formic acid (85% purity) to minced herring co-products, stirred for 30 min at 10 rpm, followed by storage at ambient temperature (i.e. ~ 22 °C)^3^. The mixture was stirred for 10 min at 10 rpm every day; and, samples were collected at regular time points and stored in 5-ml Eppendorf tubes at -80 °C until further use. The pH of the differently treated co-products before adding formic acid was around 6.50; and, the pH of ensilaged samples was within the range of 3.43–3.79 during the studied ensilaging period.

### Determination of PV and TBARS

Around 2 g co-products or silage sample was extracted using 20 ml ice-cold chloroform:methanol (2:1 v/v) containing 0.05% w/v BHT, followed by addition of 8 ml ice-cold 0.5% NaCl, according to Lee, et al.^18^. The resulting upper (i.e. methanol:water) and lower (i.e. chloroform) phases after centrifugation at 3000 × *g* for 6 min (at 4 °C) were then used to analyze TBARS and PV, respectively, according to Schmedes and Hølmer^19^ (TBARS) as well as Undeland, et al.^20^ (PV) using a spectrophotometer (Cary 60 UV–vis, Agilent technologies, USA). Briefly, for PV analysis, 2 ml chloroform phase was mixed with 1.33 ml ice-cold chloroform:methanol (1:1 v/v), followed by addition of 33.4 µl ammonium thiocyanate and 33.4 µl freshly made iron (II) chloride solution. The mixture was then vortexed, incubated for 20 min at ambient temperature (i.e. 22 °C), and then the absorbance was read at 500 nm. For TBARS analysis, 2 ml methanol:water phase was mixed with 2 ml thiobarbituric acid (TBA) reagent, incubated at 100 °C for 30 min, followed by centrifugation at 2000 × *g* for 3 min, and then absorbance of the solution was read at 532 nm.

### Analysis of Hb changes in a trout hemolysate model system

The effect of adjusting the pH to 3.50, which is a crucial part of the ensilaging process, on the shift in Hb spectra, heme group release from Hb, and Hb precipitation was investigated using Tris buffer (0.1 M, pH 8.0) enriched with trout hemolysate to a final Hb concentration of 68.68 µmole/kg (i.e. the concentration of Hb found in herring filleting co-products). The pH of this buffer model system was then adjusted to 3.50 using formic acid (85% purity), and the system was stirred continuously at ambient temperature (i.e. 22 °C). Samples for heme release from Hb were collected at regular time points and stored directly in -80 °C for subsequent analysis. Samples for the shift in Hb spectra were prepared by centrifugation at 16,000 × *g* for 5 min (4 °C), followed by scanning of the supernatants in the wavelength range of 300–700 nm using a spectrophotometer (Cary 60 UV–vis, Agilent technologies, USA). A portion of these supernatants were also immediately stored in -80 °C for subsequent protein content analysis.

### Determination of heme release from Hb

Heme group release from Hb was measured according to a method described by Maestre, et al.^21^ using ISOLUTE^®^ C18 100 mg/ml cartridges (Biotage, Sweden). Briefly, the cartridges were preconditioned with 2 ml methanol:water (1:1 v/v) with a constant flow of air, from a vacuum pump, passing through the cartridges throughout the experimentation. Thereafter, 1 ml sample was added to the cartridge, and, Hb was then eluted using 4 ml MQ-water, followed by passing of air for 15 min, and then free heme was eluted using 1 ml methanol. The methanol portion was then scanned in the wavelength range of 350–450 nm (Cary 60 UV–vis, Agilent technologies, USA), and the heme group concentration was calculated using a hemin standard (Sigma-Aldrich, Netherlands).

### Determination of protein content

Protein content in the supernatant of the trout Hb model system, was measured according to Lowry, et al.^22^ using a Bio-Rad DC protein assay kit and bovine serum albumin (Sigma) as standard. Briefly, 500 µl of reagent A was added to 100 µl of diluted sample, vortexed for 10 s, and then 4 ml of reagent B was added, and vortexed. The mixture was then incubated at ambient temperature (i.e. 22 °C) for 15 min, and, the absorbance was read at 750 nm.

### Determination of total phenolics and carnosic acid in antioxidants

Total phenolics content (TPC) of Duralox MANC-213 and the used rosemary extract was analyzed according to the Folin–Ciocalteu method^23^ with slight modifications as described by Trigo, et al.^24^. Briefly, 0.1 g sample was mixed with 10 ml extraction solvent (methanol:water + trifluoroacetic acid; 70%:30% v/v + 1% v/v), vortexed for 30 s and centrifuged at 5000 × *g* for 5 min. Thereafter, 50 µl supernatant was mixed with an equal volume of Folin–Ciocalteu reagent, followed by addition of 1 ml Na_2_CO_3_ (75 g/L) and 1.4 ml MilliQ-water. The mixture was then vortexed for 10 s, incubated for 1 h in darkness at room temperature (i.e. 22 °C), and then the absorbance was read at 750 nm. Gallic acid (Janssen Chimica, Belgium) was used as the standard for the external calibration curve (range: 25–120 µg/ml), and TPC was expressed as mg gallic acid equivalent/g sample.

Carnosic acid content of the same samples was analyzed by HPLC according to the method described by Zhang, et al.^25^ with slight modifications. Briefly, 0.1 g sample was dissolved in 10 ml methanol, vortexed for 30 s, centrifuged at 2000 × *g* for 3 min, and the resulting supernatant was used for HPLC analysis. Chromatographic separation of carnosic acid was performed on a HPLC column (Inertsil^®^ ODS-3, 3 µm, 150 × 3 mm, GL Sciences Inc., Japan) at 40 °C column temperature with 0.5 ml/min flow rate for 14 min run time and 10 µl sample injection volume. Eluents used were; (A) 1% acetic acid in Milli-Q water, and (B) 1% acetic acid in methanol. Eluent gradient conditions used were: 10% A and 90% B at 0 min, followed by gradual increase of A to 64% over 10 min, and then gradual decrease of A to 10% from 12 min; the latter ratio was maintained until the end of the run. Detection was performed using a UV detector at 280 nm wavelength, and carnosic acid standard (Sigma Aldrich 91209, Supelco, analytical standard) was used for peak detection and quantification with an external calibration curve (range: 10–750 µg/ml).

### Incubation of herring filleting co-products in different treatment solutions

Herring filleting co-products were subjected to pre-incubation, for either 30 s or 20 min or 2 h, in different treatment solutions as mentioned in Table 1. The treatment solutions were prepared in tap water and stored in cold room (4 °C) before being used in trials 1–4. The ratio of treatment solution to herring filleting co-products was 5:1 v/w in trials 1–3, and 2:1 v/w or 3:1 v/w in trial 4. Incubation treatments were performed by incubating 1 kg co-products in treatment solutions, followed by draining off the solution using a stainless-steel fine strainer. The co-products were then minced and ensilaged for 7 days as described earlier. The relative percentage (%) inhibition of TBARS, compared to the non-treated control, was calculated using the formula below:

$${\text{Inhibition of TBARS}}\, = \,\left( {\left( {{\text{TBARS non-treated control day X}} {-} {\text{TBARS treatment day X}}} \right)/{\text{TBARS non-treated control day X}}} \right) \, \times { 1}00.$$

### Ionic strength determination

Co-products or silage samples were diluted three times using MQ-water, centrifuged at 2000 × *g* for 3 min, and the supernatants were used for ionic strength measurement using a conductivity meter (CDM210, MeterLab^®^, Radiometer Analytical, France). NaCl was used as the standard; and, the ionic strength was expressed as percentage (%) NaCl equivalents.

### Determination of α-tocopherol

α-Tocopherol content in co-products and silage samples were analyzed according to Larsson and Undeland^26^ with modifications as described by Sajib and Undeland^11^. Briefly, 8 ml chloroform extract from a chloroform:methanol extraction (as described earlier) was dried under N_2_, followed by dilution in 1 ml methanol, centrifugation at 2000 × *g* for 3 min, whereafter the supernatant was analyzed using HPLC with fluorescence detection. Quantification was done using α-tocopherol standard (Sigma-Aldrich, USA).

### Statistical analysis

Results were expressed as mean values (n = 2 or 3) ± standard error of the mean (SEM). ANOVA analysis with Tukey Honest Significant Differences (HSD) test was performed on R software environment (https://www.r-project.org/), and significant differences were accepted at *p* < 0.05.

## Results and discussion

### Role of Hb in lipid oxidation development during ensilaging

**Figure 1:**
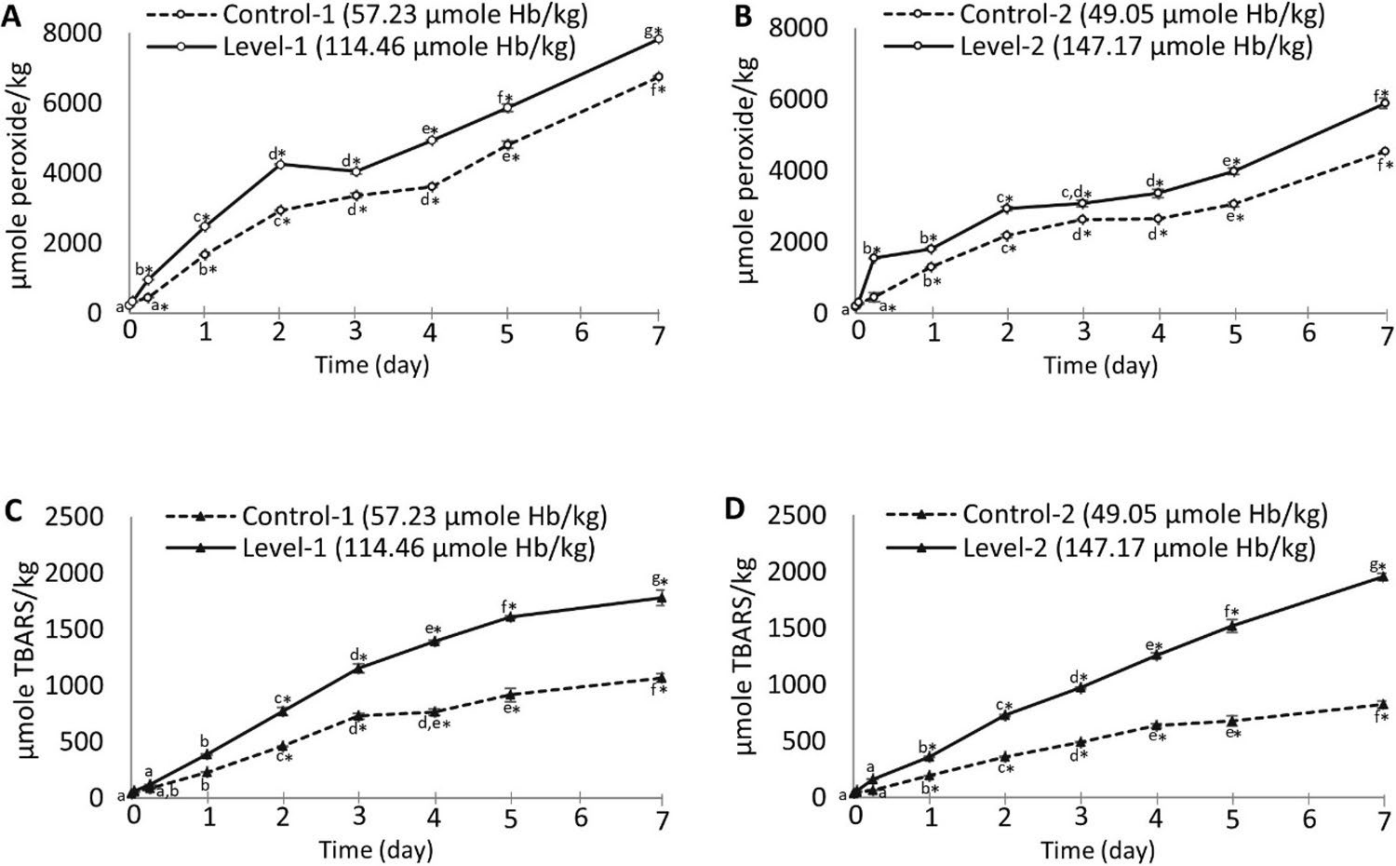
Effect of Hb fortification on PV (A-B) and TBARS (C-D) development during ensilaging of herring filleting co-products at 22 °C. Controls refer to samples without Hb fortification, and time point zero (0) refers to the sample before ensilaging. Filleting co-products from batch-2 was used in this experiment, and their endogenous Hb-level was 68.68 µmole Hb/kg. Control-1 and 2 contained 57.23 and 49.05 µmole Hb/kg silage, respectively; and, level-1 and 2 contained 114.46 and 147.17 µmole Hb/kg silage, respectively. Star (*) sign represents significant (*p* < 0.05) difference between the control and Hb-fortified samples at specified time points; and, different lower-case letters along the same line denote significance (*p* < 0.05) difference. Results are expressed as mean ± SEM (n = 2).

The role of Hb in PV and TBARS development during ensilaging of herring filleting co-products is shown in Fig. 1. Significantly (*p* < 0.05) increased PV´s were noticed in Hb-fortified samples after 6 h of ensilaging, compared to the controls (Fig. 1A,B). The PV´s of both Hb-fortified samples then remained around 500–1000 µmol/kg higher than the control samples throughout the whole studied period, despite the fact that the Hb levels were very different. At the end of the ensilaging, the relative differences in PV´s between two- and threefold Hb-fortified samples and their controls were 15.93% and 29.84%, respectively; however, a higher absolute level of PV was noticed in the twofold Hb-fortified samples, compared to the threefold fortified one, i.e. 7826.58 vs. 5887.89 µmole peroxide/kg, respectively.

In case of TBARS, significantly (*p* < 0.05) higher levels were noticed after 2 days and 1 day of ensilaging in 2- and threefold Hb-fortified samples, respectively, compared to the controls, and the difference between fortified and control samples gradually increased throughout the studied period (Fig. 1C,D). Contrary to PV-data, a higher level of TBARS was noticed in the sample with threefold Hb-level, compared to the one with twofold Hb-level, i.e. 1954.96 vs. 1777.02 µmole TBARS/kg, respectively, after 7 days of ensilaging. The relative difference to the control at the end of the ensilaging was also larger for the former sample, i.e. 137.60% vs. 66.51% for the sample with threefold vs. twofold Hb-level, respectively.

The fact that both PV and TBARS values increased significantly (*p* < 0.05) over time in all samples was in line with our earlier findings^11^, confirming that herring co-product ensilaging is a system with high sensitivity to oxidation, despite the gradual formation of short-chain, potentially antioxidative, peptides^3^. Several earlier studies have shown that fish protein-derived peptides are efficient chelators for low molecular weight (LMW) iron^27^; however, their inability to prevent oxidation in herring silage points at heme-bound iron or other compounds as being of higher relevance as pro-oxidants. The importance of Hb/heme was evident in this study as an elevated level of Hb resulted in an increased level of TBARS. The effect on PV was much smaller, illustrating the ability of Hb and heme to react with lipid hydroperoxides generating ferryl heme protein radicals, lipid radicals and hydroxyl radicals^28^, altogether preventing a significant buildup of peroxides. Earlier studies in washed cod mince models (pH 6.5–6.8) have shown a strikingly constant ratio between maximum TBARS values reached and the Hb levels added, i.e. 13.6 ± 4.8 µmole TBARS/µmole Hb tetramer (range 6.4–24, *n* = 15)^6,15^. In this present study, where Hb was both endogenous and added, the ratio was however surprisingly similar, i.e. 16.05 ± 2.25 µmole TBARS/µmole Hb tetramer (range 13.28–18.64, *n* = 8), even though this system was acidified and contained hydrolyzed proteins. This implies that Hb/heme strongly controls oxidation development both when present in situ and when added, and its quantification could provide a basis for predicting the subsequent degree of oxidation in both fish muscle and silage derived thereof.

### Effect of ensilaging on Hb change—the trout hemolysate model system

**Figure 2:**
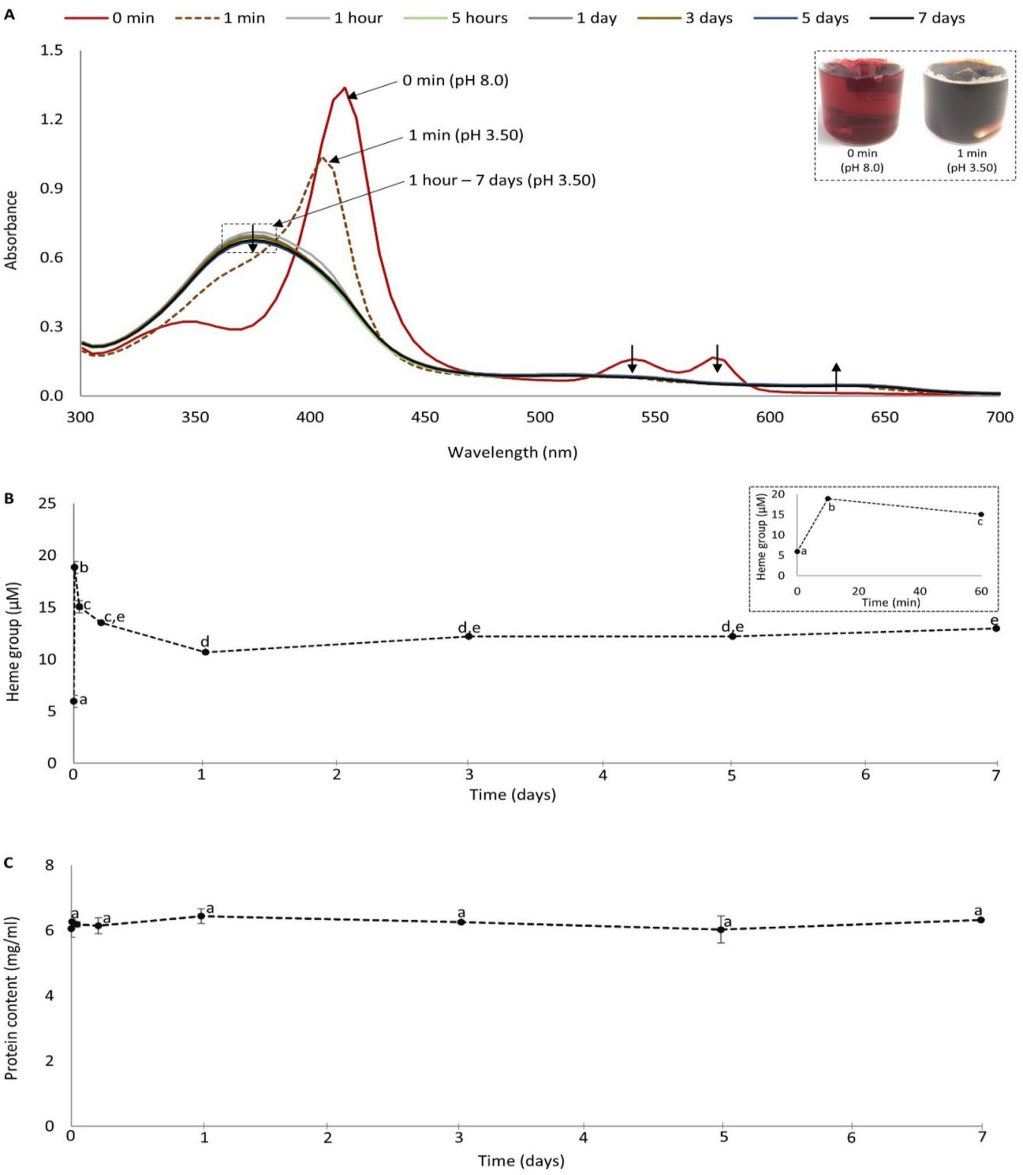
Effect of adjusting a solution of trout Hb (68.68 µmole/kg) in 0.1 M Tris buffer from pH 8.0 to pH 3.50 on shift in Hb absorption spectra **(A)**, heme group release **(B)**, and soluble protein content **(C)** over time at 22 °C. The soluble protein content was determined in the supernatant after centrifugation of the sample at 16,000 × *g* for 5 min (4 °C). The insets in **(A,B)** show the changes in Hb color from red to dark brown and heme group release from the Hb, respectively, upon adjusting pH from 8.0 to 3.50. Time point zero (0) refers to a sample before adjusting pH to 3.50 (i.e. pH 8.0). Different lower-case letters denote significance (*p* < 0.05) difference. Results are expressed as mean ± SEM (n = 2).

To further understand the role of Hb in lipid oxidation during ensilaging, we used a simple trout hemolysate model system and simulated ensilaging conditions by adjusting the pH to 3.50, and then followed the shift in Hb spectra (Fig. 2A), heme group release from Hb (Fig. 2B), and Hb precipitation (Fig. 2C) over time. As can be seen in Fig. 2A, the trout hemolysate had a bright red color before ensilaging, reflecting the oxygenated state of Hb (i.e. the oxyHb form), which is also visible from its distinguishable peaks around 415, 540, and 580 nm. However, the bright red color turned into brown immediately after adjusting the pH to 3.50, illustrating the change from oxyHb to metHb, which was also evident by the disappearance of peaks around 540 and 580 nm, a shift of the peak around 415 nm to around 405 nm, as well as development of a peak around 630 nm. A peak around 375 nm also appeared in the spectra, which was probably due to release of the heme group from Hb. The decreasing intensity of this peak over time most likely reflects gradual heme group degradation with subsequent release of free iron. The significant (*p* < 0.05) release of the heme group from Hb immediately after adjusting the pH to 3.50 and its gradual degradation over time was also supported by the analysis of heme groups present in the hemolysate-buffer model system (Fig. 2B). Hargrove, et al.^29^ reported that the release of heme from Hb is around 60 times faster from metHb, compared to oxyHb and deoxyHb, which thus supports our observed increase in heme group release immediately after the acid-induced shift in Hb spectra from oxyHb to metHb. The exposure of the heme group to the surrounding environment stimulates autoxidation and subsequent heme-loss^30^. Most likely, these changes explain the rapid increase in PV, followed by an increased TBARS value, which took place during ensilaging of herring filleting co-products (Fig. 1). The protein content in the hemolysate-buffer model system remained constant throughout the studied time period (Fig. 2C), suggesting that Hb did not precipitate during ensilaging.

### Effect of pre-incubating the co-products in water or salt solutions with and without antioxidants on lipid oxidation

**Figure 3:**
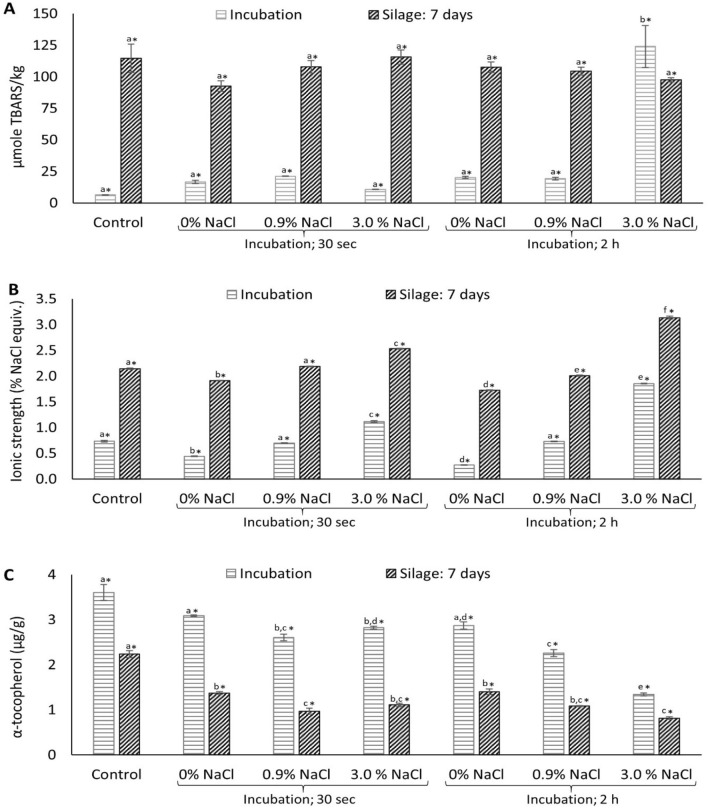
Effect of pre-incubating the co-products for 30 s or 20 min in TBARS **(A)**, ionic strength **(B)**, and α-tocopherol **(C)** immediately after incubation and after 7 days of ensilaging. Control refers to sample without any treatment. Filleting co-products from batch-1 was used in this trial. Star (*) sign represents significant (*p* < 0.05) difference between incubated and ensilaged samples subjected to the same treatment; and, different lower-case letters among incubation treatments or silages denote significance (*p* < 0.05) difference between treatments. Results are expressed as mean ± SEM (n = 3).

To minimize Hb-mediated lipid oxidation during ensilaging, we investigated the possibility of pre-incubating the herring co-products in different treatment solutions. The hypothesis was that removing Hb from the outer layer of the co-products, with or without covering the surface by antioxidants, could possibly minimize lipid oxidation. In our first trial, we incubated the co-products in physiological salt solution (i.e. 0.9% NaCl) to prevent the lysis of erythrocytes^31^. In addition, we also investigated the possibility of using 3.0% NaCl to simulate seawater, which is traditionally used during pre-storage of herring prior to processing. Controls were incubation in tap water (i.e. 0% NaCl) and non-treated. Incubating herring co-products in 0.9% NaCl for 20 min has previously been shown to remove 6.6 – 18.0% Hb; the exact amount varied with the specific co-product part i.e. 6.6, 10.3, 17.9, and 18.0% from fin, head, backbone, and residuals, respectively^5^. However, in this study, no significant (*p* > 0.05) differences in TBARS were noticed after incubating the co-products for 30 s or 2 h in water or salt solutions, except for the 2 h incubation in 3.0% NaCl which resulted in a significant (*p* < 0.05) increase in TBARS, compared to the non-treated control (Fig. 3A). This was possibly due to the hypertonic nature of 3.0% NaCl, causing lysis of erythrocytes^32^, as well as the known pro-oxidative ability of NaCl in muscle tissue^33^. Figure 3B illustrates the actual change in ionic strength as a result of the incubations, and it can be seen that co-products incubated in 3.0% NaCl for 30 s or 20 min obtained an ionic strength of around 1.11 and 1.85% NaCl-equivalents, respectively. Analysis of the endogenous antioxidant α-tocopherol in the co-products revealed that incubating in 0.9% and 3.0% NaCl resulted in a significantly (*p* < 0.05) lower level of α-tocopherol content, compared to the non-treated control (Fig. 3C). Thus, the slight removal of pro-oxidative Hb was counteracted by a simultaneous loss of endogenous α-tocopherol during the incubations, which otherwise could have provided antioxidative effect by donating a hydrogen atom to free radicals such as L● or LOO● to disrupt the propagation process and ultimately reduce the formation of hydroperoxides^34^.

Increased TBARS values were noticed after 7 days of ensilaging in all treatments, and there were no significant (*p* > 0.05) effects from different treatments (Fig. 3A). Further, a significant (*p* < 0.05) increase in the ionic strength was noticed in silages (Fig. 3B), compared to the incubated co-products, which was possibly due to the addition of acid. The latter was confirmed by an increase in the ionic strength immediately after adding acid to the minced co-products, which later remained relatively constant throughout the studied ensilaging period (see supporting information; Fig. 1). Thus, in accordance with Fig. 1, these data confirmed that the ensilaging process itself, partly due to the acidic conditions, promotes lipid oxidation, which is also apparent from a significant (*p* < 0.05) consumption of α-tocopherol after 7 days of ensilaging (Fig. 3C). The negative correlation between TBARS and α-tocopherol level over time was in agreement with our previous study^11^.

**Figure 4:**
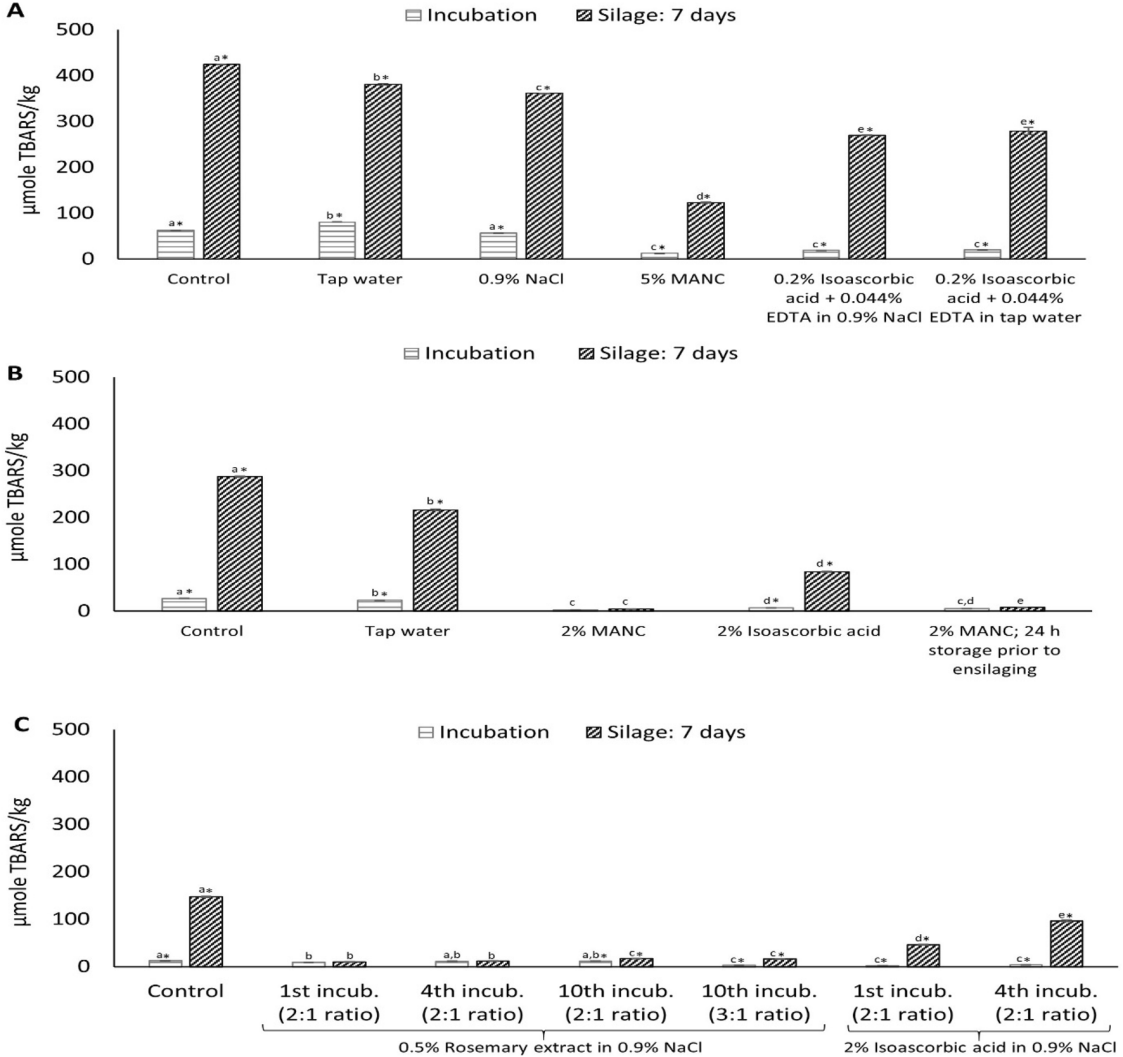
Effect of pre-incubating the co-products in water, 0.9% NaCl or antioxidant solutions for 20 min **(A,B)**, and reusing the solution for 4 or 10 treatments, lowering the solution to co-products ratio from 5:1 to 3:1 or 2:1, shortening the treatment time from 20 min to 30 s, and, using a rosemary extract alone **(C)** on TBARS development immediately after incubation and after 7 days of ensilaging. Control refers to sample without any treatment. Filleting co-products from batch 2, 3, and 4 were used for trials 2, 3, and 4 as shown in panel **(A–C)**, respectively. Star (*) sign represents significant (*p* < 0.05) difference between incubation and silage samples within the same treatment; and, different lower-case letters among incubation treatments or silages denote significance (*p* < 0.05) difference between treatments. Results are expressed as mean ± SEM (n = 3). MANC: Duralox MANC-213; *incub.* incubation.

Based on the outcomes of our first trial, antioxidants were added to the treatment solutions to compensate for the loss of endogenous α-tocopherol and to add extra protection. Incubating the co-products in solutions made from 0.2% isoascorbic acid with 0.044% EDTA in tap water or in 0.9% NaCl for 20 min significantly (*p* < 0.05) lowered the TBARS values both immediately after the incubation and after 7 days of ensilaging, compared to the non-treated control, incubated only in tap water or 0.9% NaCl (Fig. 4A). Isoascorbic acid provides antioxidative effect by scavenging free radicals and reducing hypervalent iron^35^, while EDTA works by chelating metal ions like ferrous and ferric iron^36^. The commercial antioxidant mixture Duralox MANC-213 at 5%, gave the same low TBARS values immediately after incubation, but provided a significantly (*p* < 0.05) stronger inhibitory effect after 7 days of ensilaging (Fig. 4A). Among the treatments used, Duralox MANC-213 provided the highest TBARS inhibition, compared to the non-treated control, both directly after incubation and after 7 days of ensilaging, i.e., 79.50 and 70.95%, respectively (Table 2). This was probably because of its multiple ingredients with rosemary extract being the major one, further to tocopherols, ascorbic acid and citric acid, providing synergistic effects^37^. The rosemary extract of Duralox MANC-213 contains many phenolic compounds including rosmarinic acid, carnosic acid, and carnosol; the latter two which have been reported as the most active compounds of rosemary extract^38^. The same authors also reported that both carnosic acid and carnosol provided better inhibitory effect at pH 4.0 than at pH 7.0, in a corn oil-in-water emulsion system oxidized at 60 °C for 4 days, which is probably due to their stability, better reducing capacity, and partitioning either in the oil phase or in the oil–water interface at lower pH values^38^. Our own analyses revealed a carnosic acid level of 1.55 ± 0.06 mg/g Duralox-MANC-213 and a TPC of 69.63 ± 2.29 mg gallic acid eq/g; the latter indicating there were a lot of phenolic compounds beyond the carnosic acid. Further, Duralox MANC-213 contains both hydrophilic (e.g. ascorbic acid, citric acid and aqueous rosemary-derived compounds) and lipophilic compounds (e.g. tocopherol and rosemary-derived lipophilic compounds), which possibly aided its partitioning both into the oil phase and oil–water interface under acidic ensilaging conditions, supporting its strong antioxidative protection during ensilaging.

**Table 2:** Relative percentage (%) inhibition of TBARS, compared to the non-treated control.

Trial	Treatments	Incubation	Silage
1	0% NaCl (5:1 ratio); 30 s	−162.77	19.16
	0.9% NaCl (5:1 ratio); 30 s	−233.19	5.89
	3.0% NaCl (5:1 ratio); 30 s	−68.54	−0.83
	0% NaCl (5:1 ratio); 2 h	−217.78	6.06
	0.9% NaCl (5:1 ratio); 2 h	−204.00	8.85
	3.0% NaCl (5:1 ratio); 2 h	−1852.74	14.91
2	Tap water (5:1 ratio); 20 min	−28.99	10.26
	0.9% NaCl in Tap water (5:1 ratio); 20 min	9.32	14.84
	5% MANC in Tap water (5:1 ratio); 20 min	79.50	70.95
	0.2% isoascorbic acid with 0.044% EDTA in 0.9% NaCl (5:1 ratio); 20 min	69.26	36.39
	0.2% isoascorbic acid with 0.044% EDTA in tap water (5:1 ratio); 20 min	68.23	34.21
3	Tap water (5:1 ratio); 20 min	15.07	24.69
	2% MANC in tap water (5:1 ratio); 20 min	89.14	98.53
	2% isoascorbic acid in tap water (5:1 ratio); 20 min	74.26	70.76
	2% MANC in tap water (5:1 ratio); 20 min; prolonged storage at 4 °C for 24 h	80.24	97.21
4	0.5% rosemary extract in 0.9% NaCl; 1st incubation (2:1 ratio); 30 s	30.75	93.07
	0.5% rosemary extract in 0.9% NaCl; 4th incubation (2:1 ratio); 30 s	14.65	91.66
	0.5% rosemary extract in 0.9% NaCl; 10th incubation (2:1 ratio); 30 s	10.95	88.26
	0.5% rosemary extract in 0.9% NaCl; 10th incubation (3:1 ratio); 30 s	74.76	89.01
	2% isoascorbic acid in 0.9% NaCl; 1st incubation (2:1 ratio); 30 s	80.61	68.56
	2% isoascorbic acid in 0.9% NaCl; 4th incubation (2:1 ratio); 30 s	68.07	34.69

Negative and positive values denote percentage increase and decrease, respectively, in TBARS values compared to the non-treated control.

MANC: Duralox MANC-213; 5:1, 2:1 and 3:1 refer to the solution to co-product ratios used in different treatments.

In the next trial (i.e. trial 3) the amount of Duralox MANC-213 in the incubation solution was lowered from 5 to 2% and it was also compared with 2% isoascorbic acid. Further, the effect on TBARS from pre-storing the co-products at 4 °C for 24 h after incubation was investigated to simulate a case where co-products would need to be stored/transported to another plant before being ensilaged. Results show that incubating the co-products in 2% Duralox MANC-213, with or without pre-storage at 4 °C for 24 h, was sufficient to significantly (*p* < 0.05) inhibit TBARS development compared to the tap water-treated control and the 2% isoascorbic acid-treated sample, both immediately after the incubation and after 7 days of ensilaging (Fig. 4B). It also gave better TBARS inhibition compared to the incubation in 5% Duralox MANC-213 (Table 2). This could however partly be due to high-quality starting raw material with less pre-formed oxidation products used in this trial. Even though there was a very slight, but still significant (*p* < 0.05), increase in TBARS in the pre-stored co-products after 7 days of ensilaging, our data suggest that pre-incubated co-products can be stored for some time prior to ensilaging without compromising the oxidative stability of the co-products or the silage. However, microbial stability during this pre-storage time without acidification should be considered and requires further investigation. Trial 3 also revealed that 2% isoascorbic acid alone, in relative terms, was as efficient as 0.2% isoascorbic acid with 0.044% EDTA in preventing oxidation during the incubation and subsequent ensilaging (Fig. 4A vs B, and Table 2). Isoascorbic acid alone provided 70.76% TBARS inhibition after 7 days ensilaging, while the combination with EDTA provided 34.21% TBARS inhibition (Table 2). This illustrates that LMW-iron is of minor importance as a pro-oxidant in herring silage, rather heme-bound iron is the dominant pro-oxidant.

In the last trial, (i.e. trial 4), we investigated the possibilities of reusing the antioxidant solution for up to 10 incubation treatments, lowering the solution to co-products ratio from 5:1 to 3:1 or 2:1, shortening the treatment time from 20 min to 30 s, and using a rosemary extract alone in TBARS inhibition (Fig. 4C). There were no significant (*p* > 0.05) differences in the immediate TBARS-values after reusing the solution 4 and 10 times, compared to using it in one incubation treatment. Similarly, there were no significant (*p* > 0.05) differences in TBARS developments after 7 days of ensilaging, except that a slight but significant (*p* < 0.05) increase in TBARS was noticed after reusing the solution for 10 times. The use of a 3:1 solution to co-products ratio resulted in a significantly (*p* < 0.05) lower TBARS value immediately after incubation, compared to the 2:1 ratio, however, there was no significant (*p* > 0.05) difference after 7 days of ensilaging. Further, the use of 0.5% rosemary extract and 30 s incubation time were very effective and significantly (*p* < 0.05) inhibited TBARS development in silage, compared to the non-treated control and to 2% isoascorbic acid. It was also evident that among all the treatments from trial 1–4, both Duralox MANC-213 and rosemary extract, in relative terms, provided the highest TBARS inhibitory effects after 7 days of ensilaging, i.e., inhibitions were 70.95–98.53% and 88.26–93.07%, respectively (Table 2). This confirms that rosemary extract remains stable and active even under the acidic ensilaging conditions, and it can constitute a cheaper option than Duralox MANC-213, also requiring less labelling. The carnosic acid level of the rosemary extract was much higher than in Duralox MANC-213 (57.84 ± 1.54 vs. 1.55 ± 0.06 mg/g), explaining its high activity even in the absence of other antioxidants such as those present in the Duralox MANC-213 mixture (i.e. ascorbic acid, citric acid and tocopherol). Also, its TPC was higher, but to a much smaller extent (102.53 ± 0.04 vs 69.63 ± 2.29 mg gallic acid eq/g), revealing that other phenolics than carnosic acid played a dominant role in Duralox MANC-213.

The last trial also yielded several pieces of information which can make the incubation technology more scalable, provided that the used antioxidant is effective in inhibiting TBARS during ensilaging, e.g. (i) the incubation time could be shortened, (ii) the same solution could be reused for several incubation treatments, and (iii) the ratio between solution and co-products could be lowered. However, it is very important to also consider that just as we can remove a portion of Hb during the incubations, we are also losing some proteolytic enzymes by such treatments, which slightly reduced the protein hydrolysis rate during ensilaging (see supporting information; Fig. 2). We believe though that the advantage of using incubation treatments to minimize lipid oxidation during ensilaging outweighs the slightly reduced rate of protein hydrolysis as the final silage will have a much higher quality. Further, the proteins lost into the incubation solution can be recovered by using techniques such as flocculation combined with flotation or ultrafiltration, as reported elsewhere^39–41^, ensuring as complete use as possible of the herring raw material.

## Conclusion

The role of Hb in lipid oxidation development during ensilaging of herring filleting co-products was here reported for the first time. The presence of extra added Hb in herring co-products resulted in largely increased levels of TBARS during ensilaging, but only slightly increased levels of PV, confirming the ability of Hb/heme to mediate oxidation mainly by decomposing peroxides. A trout hemolysate model system confirmed that the native oxyHb immediately changed to metHb upon adjusting the pH to that needed for ensilaging (i.e. pH 3.50), which facilitated heme group release from the Hb. It is therefore suggested that the likely mechanism by which Hb promoted lipid oxidation during ensilaging was via heme-mediated peroxide cleavage. To minimize Hb-mediated lipid oxidation, the most promising strategy, as shown in this study, was pre-incubation of the herring co-products for 30 s in 2–3 volumes of solutions containing 0.5% rosemary extract, which effectively inhibited TBARS both in the co-products and in the final silage. The active compounds of rosemary extract, i.e. carnosic acid and carnosol, were most probably stable even under the acidic conditions used in ensilaging and thus provided the highest inhibitory effect. The fact that the same incubation solution could be reused for up to 10 times without losing its activity paves the way for a scalable and easily applicable technology to stabilize the herring filleting co-products per se, but also a value-added product derived from them. It is foreseen that high-quality fish silages with low oxidation levels can have a future market not only as feed ingredients, but also as peptide-rich food ingredients.

## Supplementary Information

Supplementary Information.
