# Supplementary material for: Hemoglobin-mediated lipid oxidation of herring filleting co-products during ensilaging and its inhibition by pre-incubation in antioxidant solutions
Source: Sci Rep. 2021 Sep 30;11:19492. doi: 10.1038/s41598-021-98997-4 (PMC8484477; doi:10.1038/s41598-021-98997-4)
Supplement: Supplementary file 1 — Supplementary Information. [file 41598_2021_98997_MOESM1_ESM.docx]

**Supporting information**

**Hemoglobin-mediated lipid oxidation of herring filleting co-products during ensilaging and its inhibition by pre-incubation in antioxidant solutions**

Mursalin Sajib*, Haizhou Wu, Rikard Fristedt, and Ingrid Undeland

Food and Nutrition Science, Department of Biology and Biological Engineering, Chalmers University of Technology, SE-41296 Gothenburg, Sweden

*Corresponding author

Tel: +4631 772 68 63

E-mail: mursalin@chalmers.se

**Table 1. TBARS values of controls and Hb-fortified silages**

| Treat. | µmole TBARS/kg silage (mean ± SEM) | | | | | | | | |
| --- | --- | --- | --- | --- | --- | --- | --- | --- | --- |
|  | **0 h** | **1 h** | **6 h** | **1 d** | **2 d** | **3 d** | **4 d** | **5 d** | **7 d** |
| Control-1 | 34.55 ± 0.29 | 56.54 ± 0.18 | 82.55 ± 0.88 | 227.87 ± 1.77 | 458.03 ± 4.22 | 726.57 ± 26.15 | 762.84 ± 28.90 | 914.72 ± 59.32 | 1067.22 ± 38.89 |
| Control-2 | 30.75 ± 0.09 | 46.29 ± 0.87 | 63.60 ± 0.12 | 190.06 ± 1.35 | 358.72 ± 4.85 | 486.93 ± 5.27 | 634.73 ± 19.95 | 676.31 ± 47.49 | 822.77 ± 31.34 |
| Level-1 | 36.10 ± 0.25 | 58.54 ± 0.15 | 116.05 ± 1.40 | 385.28 ± 5.74 | 767.91 ± 32.91 | 1151.01 ± 41.03 | 1390.39 ± 11.30 | 1607.25 ± 12.60 | 1777.03 ± 68.36 |
| Level-2 | 36.00 ± 0.17 | 57.44 ± 0.41 | 159.73 ± 2.70 | 357.58 ± 4.96 | 724.65 ± 6.88 | 969.86 ± 8.84 | 1259.49 ± 17.91 | 1519.15 ± 58.35 | 1954.97 ± 31.52 |

Control-1 and -2 contained 57.23 and 49.05 µmole Hb/kg, respectively; and, level-1 and -2 contained 114.46 and 147.17 µmole Hb/kg, respectively. Treat.: treatment.

**Table 2. TBARS values of different incubation trials**

| Trial | Treatments | µmole TBARS/kg Silage (mean ± SEM) | |
| --- | --- | --- | --- |
|  |  | Incubation | Silage |
| 1 | Control | 6.35 ± 0.02 | 114.64 ± 11.36 |
|  | 0% NaCl (5:1 ratio); 30 sec | 16.69 ± 1.26 | 92.68 ± 4.21 |
|  | 0.9% NaCl (5:1 ratio); 30 sec | 21.16 ± 0.16 | 107.89 ± 4.93 |
|  | 3.0 % NaCl (5:1 ratio); 30 sec | 10.70 ± 0.08 | 115.60 ± 5.66 |
|  | 0% NaCl (5:1 ratio); 2 h | 20.18 ± 0.94 | 107.70 ± 4.22 |
|  | 0.9% NaCl (5:1 ratio); 2 h | 19.30 ± 1.16 | 104.50 ± 3.12 |
|  | 3.0 % NaCl (5:1 ratio); 2 h | 124.00 ± 16.72 | 97.55 ± 1.76 |
| 2 | Control | 62.23 ± 0.37 | 423.80 ± 0.45 |
|  | Tap water (5:1 ratio); 20 min | 80.27 ± 0.38 | 380.32 ± 1.74 |
|  | 0.9% NaCl in Tap water (5:1 ratio); 20 min | 56.43 ± 0.22 | 360.91 ± 0.71 |
|  | 5% MANC in Tap water (5:1 ratio); 20 min | 12.76 ± 0.03 | 123.12 ± 0.97 |
|  | 0.2% isoascorbic acid with 0.044% EDTA in 0.9% NaCl (5:1 ratio); 20 min | 19.13 ± 0.11 | 269.58 ± 0.25 |
|  | 0.2% isoascorbic acid with 0.044% EDTA in tap water (5:1 ratio); 20 min | 19.77 ± 0.19 | 278.81 ± 8.07 |
| 3 | Control | 27.20 ± 0.44 | 287.00 ± 1.03 |
|  | Tap water (5:1 ratio); 20 min | 23.10 ± 0.13 | 216.15 ± 1.43 |
|  | 2% MANC in tap water (5:1 ratio); 20 min | 2.95 ± 0.15 | 4.22 ± 0.14 |
|  | 2% Isoascorbic acid in tap water (5:1 ratio); 20 min | 7.00 ± 0.17 | 83.93 ± 1.14 |
|  | 2% MANC in tap water (5:1 ratio); 20 min; prolonged storage at 4°C for 24 h | 5.37 ± 0.03 | 8.02 ± 0.03 |
| 4 | Control | 13.03 ± 0.18 | 147.51 ± 1.23 |
|  | 0.5% Rosemary extract in 0.9% NaCl; 1st incubation (2:1 ratio); 30 sec | 9.02 ± 0.24 | 10.23 ± 0.14 |
|  | 0.5% Rosemary extract in 0.9% NaCl; 4th incubation (2:1 ratio); 30 sec | 11.12 ± 0.62 | 12.31 ± 0.18 |
|  | 0.5% Rosemary extract in 0.9% NaCl; 10th incubation (2:1 ratio); 30 sec | 11.61 ± 0.42 | 17.31 ± 0.08 |
|  | 0.5% Rosemary extract in 0.9% NaCl; 10th incubation (3:1 ratio); 30 sec | 3.29 ± 0.12 | 16.21 ± 0.22 |
|  | 2% Isoascorbic acid in 0.9% NaCl; 1st incubation (2:1 ratio); 30 sec | 2.53 ± 0.10 | 46.38 ± 0.98 |
|  | 2% Isoascorbic acid in 0.9% NaCl; 4th incubation (2:1 ratio); 30 sec | 4.16 ± 0.08 | 96.33 ± 1.63 |

MANC: Duralox MANC-213; 5:1, 2:1 and 3:1 refer to the solution to co-product ratios used in different treatments.


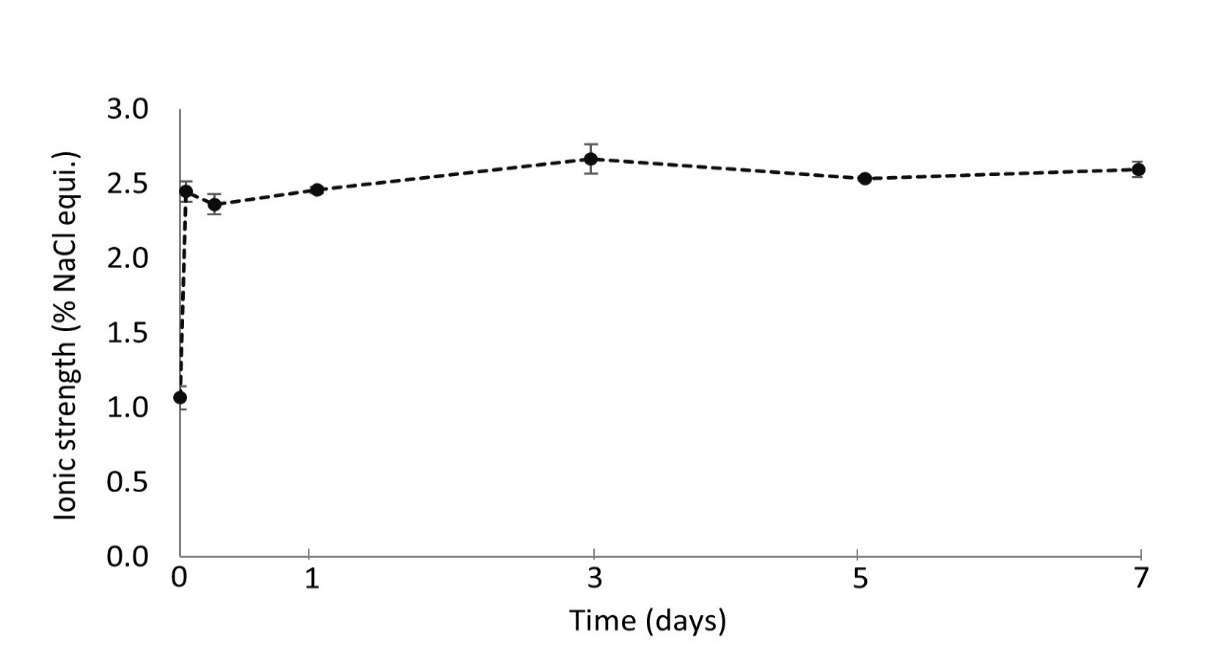


Figure 1. Changes in ionic strength during ensilaging. 0 hour refers to sample before ensilaging (i.e. before adding acid).


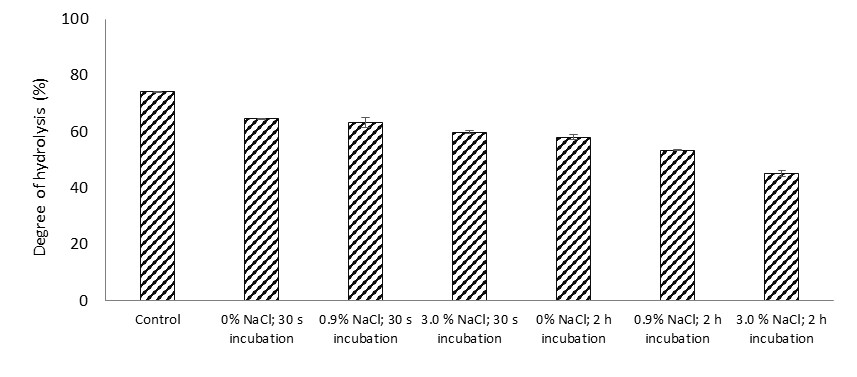


Figure 2. Effect of pre-rinsing the co-products on protein degree of hydrolysis (after 7 days of ensilaging at 22°C). Filleting co-products from batch-1 was used in this trial (i.e. trial-1 as mentioned in Table-1 in the manuscript).


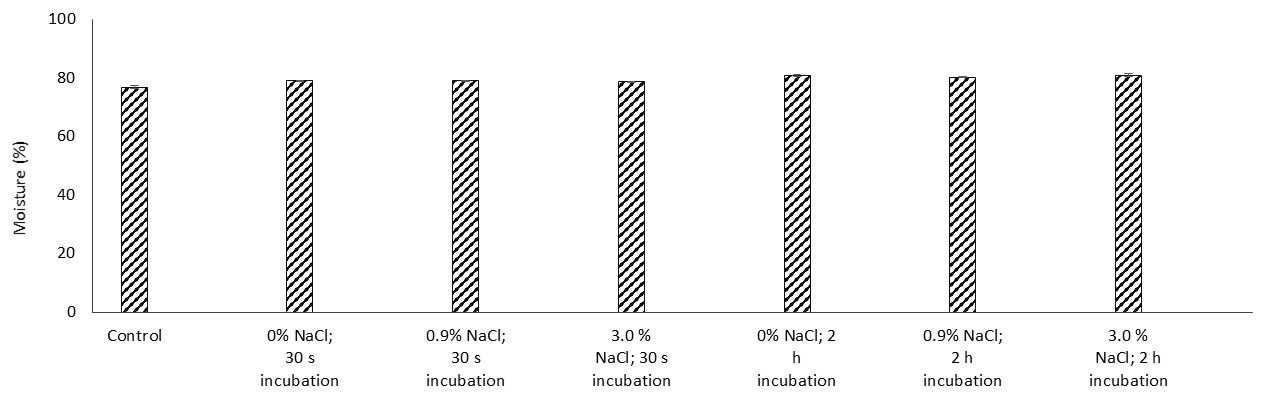


Figure 3. Moisture content after rinsing/incubating the co-products. Filleting co-products from batch-1 was used in this trial (i.e. trial-1 as mentioned in Table-1 in the manuscript).


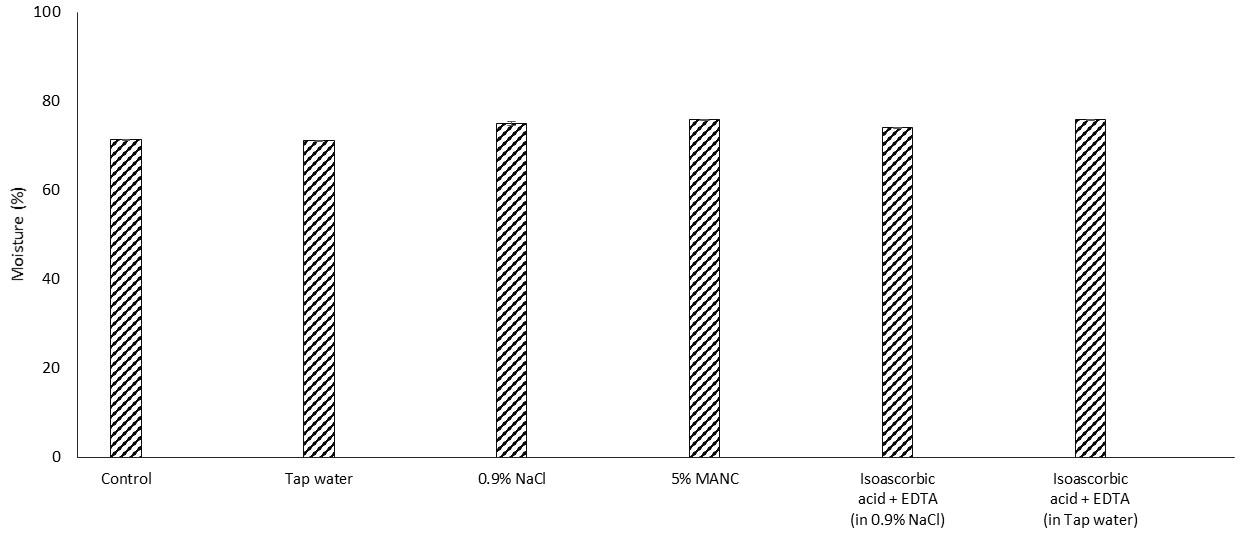


Figure 4. Moisture content after dipping the co-products for 20 min. Filleting co-products from batch-2 was used in this trial (i.e. trial-2 as mentioned in Table-1 in the manuscript).


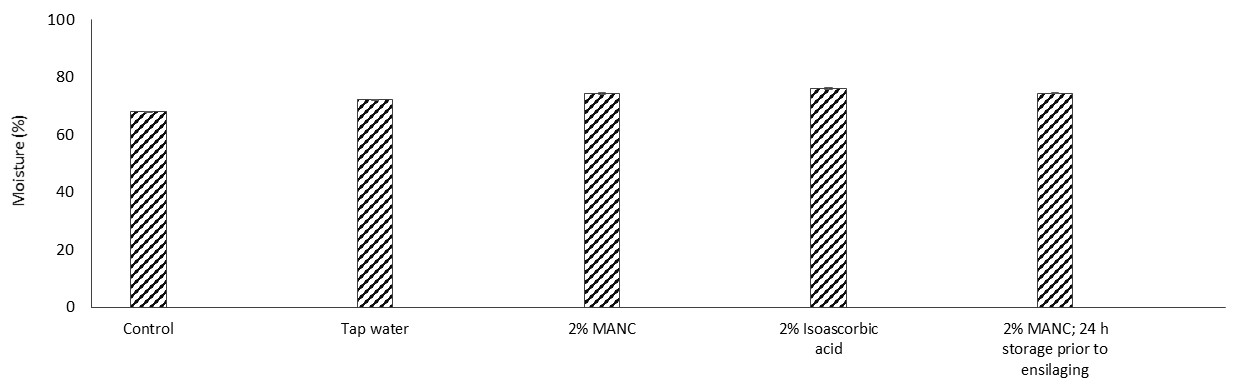


Figure 5. Moisture content after dipping the co-products for 20 min. Filleting co-products from batch-3 was used in this trial (i.e. trial-3 as mentioned in Table-1 in the manuscript).


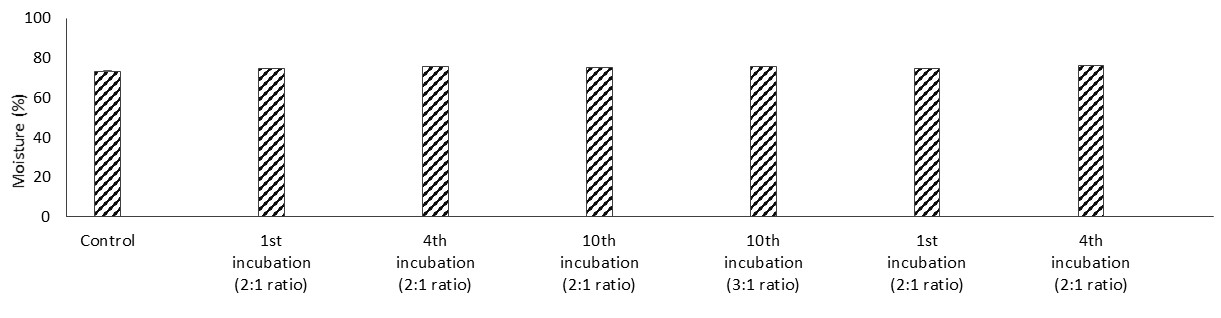


Figure 6. Moisture content after rinsing the co-products for 30 sec. Filleting co-products from batch-4 was used in this trial (i.e. trial-4 as mentioned in Table-1 in the manuscript).
